# Notes of Cases of Diphtheritic Paralysis, with Remarks

**Published:** 1884-09

**Authors:** Andrew S. Currie

**Affiliations:** Lydney, Gloucester


### NOTES OF CASES OF DIPHTHERITIC PARALYSIS, WITH REMARKS. By ANDREW S. CURRIE, M.D., Lydney, Gloucester.

In September, 1878, a boy, æt. 8, was brought to me, who was suffering from motor paralysis of the lower extremities. The loss of power was not absolute, for the child could stand when supported by some one. The symptoms had been in existence for about a week, and had supervened gradually. First, unsteadiness and a liability to stumble readily, and, afterwards, progressive difficulty in locomotion, were observed. No paralysis, or paresis, of any other part was observed, and there were no sensory troubles. At first, no history of previous illness could be obtained which might in any way account for the origin of the disorder; but on further enquiry being made, it was ascertained that six weeks prior to the development of the paretic symptoms the boy had complained of sore throat, but no particular attention had been paid to the ailment. Under the use of steel and nervine tonics the patient recovered completely.

In February last two children from the same neighbourhood were brought to me almost simultaneously. Both had suffered about the same time from sore throat, accompanied with glandular enlargement, and they had probably been infected from the same source. They first came under my observation between three and four weeks after their recovery from the throat affection.

F. W., a girl seven years of age, had been noticed to speak through her nose for a fortnight prior to my seeing her. She complained of pains in the limbs, and especially in the knees, and of great weakness. Her mother said she was almost blind.

I noted the following points: Great impairment of patellar tendon reflex on both sides. Right internal strabismus with diplopia. Pupils moderately dilated and fairly sensitive to light. No morbid appearances detected by ophthalmoscopic examination. Uncertain, straddling gait. Paretic condition of the lower limbs, but no marked impairment of sensibility. No affection of the sphincter muscles.

This child recovered rapidly and completely under treatment by steel and nux vomica.

The next case was that of a girl ten years of age. The symptoms were, briefly: Snuffling voice, amblyopia, occasional strabismus, great impairment of muscular tone generally, knee-jerk almost entirely lost. In this case, as in the preceding, the discs and retinæ were healthy. The treatment was the same, and the results were equally satisfactory.

The fourth case presented some features of special and peculiar interest. Unlike the three first, the diphtheritic affection was most pronounced. It would take up too much time and space to give a detailed history of the actual diphtheritic attack, as I shall refer hereafter to a peculiar feature in the pulse.

During the pyrexial stage the patient was treated by kairin, and the case is referred to in a communication on the therapeutics of kairin which I made to the Belfast meeting of the British Medical Association last July.

A. T. was a girl sixteen years of age, above the average height, well developed, and prior to her illness inclined to embonpoint. The diphtheritic attack came on when the patient was on a visit to her married sister. She returned to her home, which is a farm-house healthily situated on the brow of a hill, towards the end of the first week in August. At this time she was suffering from very pronounced palatal paralysis. She had a nasal snuffling voice, and fluids regurgitated through the nostrils. This latter symptom gradually passed off, and altogether the girl appeared to be convalescent, according to her friends' account; for at this time she had no medical attendance.

I saw A. T. again on the evening of the 30th of August. She was troubled with a constant flow of mucus, which her parents thought was a sign of consumption; but there was no cough. There was at this time nasal voice, but no regurgitation through the nostrils. The uvula was pendulous, insensitive and motionless, the fauces generally red and congested, and bathed with somewhat viscid mucus. Temp. 100, pulse 110. There had been considerable emaciation since my last visit to the patient, on 28th July. The chest percussion note was resonant, and auscultation revealed nothing beyond feeble respiratory murmur and slight increase of vocal resonance. Urine acid, 1026, free from albumen.

Strong soups, milk, cream and jellies, as well as a moderate amount of old port wine, were ordered, and a mixture containing nitro-hydrochloric acid and compound tincture of bark was prescribed.

31st.—Patient for the first time complained of feeling of pins and needles, chiefly in her hands and feet. She could not tell where her legs were in bed without looking. The limbs began to be tremulous, and for the first time the patient became unable to feed herself or to walk without assistance.

Sept. 2nd.—The patient stated that she was much better; her appetite had improved, and she was sleeping well at night. She had derived much comfort from hot sponging, which I had recommended. The tongue was clean and the pulse stronger, but I was much struck by the emaciation, which appeared to be progressing rapidly. The mother and sister had remarked the same thing to one another before I spoke to them of it. This emaciation was general, but was chiefly noticeable in the muscles of the arms and legs. I now noticed for the first time total absence of patellar tendon reflex. There was no ankle clonus. The chest was again carefully examined, but with purely negative results. The sister reported that the patient had been quite unable to come down stairs this morning even with assistance; but she was carried down and laid on the couch as usual at her own request.

I asked the girl to try and walk. She could not rise from the couch without assistance, though she could raise herself into a sitting posture. Supported by me on one side, she staggered across the room, throwing out her limbs in a wild ataxic manner, raising her heels to a considerable height and strongly dorso-flexing her toes. There were no lightning or other pains.

Warm bathing of the limbs, with vigorous friction, was ordered, and three minim doses of liquor strychniae were added to the bark mixture.

Next day there was no improvement. Patient complained of severe pain in lumbar portion of spine.

Sept. 4th.—On awaking she complained of severe abdominal pains. About 11 a.m. she had an alarming syncopal attack. At my visit at 11.30 a.m. the pains had almost ceased ; but the breathing was short, shallow, and hurried, and there were large mucous râles all over the chest. The paralytic condition had deepened, and the patient endeavoured in vain to sit up. Deglutition had become impossible, and a nutritive enema was not retained.

At 6.30 p.m. the patient appeared to be moribund. She lay on her side, with the saliva dribbling from her mouth and wetting the pillow. At 3 a.m. on the following morning she became very sick and was greatly relieved, but the improvement was illusory. She died about three o'clock in the afternoon of the 5th Sept. Her mind was clear to the last, and shortly before her death she gave instructions about her funeral arrangements.

Unfortunately no post-mortem could be obtained.

In regard to this last case, the point which struck me with most force was the extraordinary rapidity of emaciation. It is well known that wasting occurs more or less in diphtheria, as well as in other exhausting diseases ; but the question at once arises, Is the cause of wasting the same in all ? Is it due to tissue waste induced by pyrexia ? If this explanation might serve in the actual diphtheritic attack, it would not avail to explain the great loss of flesh in the last few days of life; for the pyrexia then was slight.

Again, we can scarcely attribute it to deficiency of aliment and non-assimilation; for the appetite was exceptionally good, and there was no evidence of any gastric disturbance. We must, moreover, eliminate another frequent cause of emaciation; viz., albuminuria. In July there was a considerable quantity of albumen in the urine; but the condition was temporary, and in the last week of life there was not a trace of albumen in the urine. Had there then been any albumen, or had there been low specific gravity of the urine, with increase in its quantity, one would have been disposed to have found in this fact the explanation required. It is now many years since Sir William Jenner pointed out that emaciation was often diagnostic of albuminoid disease.* The most probable explanation in this case is, to quote Sir James Paget, that the muscular atrophy was "due to changes in the central organs of the nervous system."† The late Professor Laycock used to teach that many cases of atrophy were instances of what he termed trophic neuroses, and he believed that there was a trophic centre, probably in the medulla oblongata. But we see cases of muscular atrophy in nervous disease limited to the spinal cord, as in anterior polio-myelitis, and there is now no doubt that the giant cells in the anterior cornua, besides representing muscular movements, or more correctly, according to Dr. Hughlings-Jackson, groups of muscular movements, govern also nutrition; and in this instance I believe that post-mortem examination would probably have revealed inflammatory and degenerative changes,

* *Medical Times and Gazette*, 1860, Vol. I., p. 465.

† *Lectures on Surgical Pathology*, 4th Edition, p. 93.

not merely in the medulla, but in the anterior columns of the cord, possibly also in the anterior nerve roots; and not only would this condition have accounted for the atrophic changes, but it would have afforded a valid explanation of the paresis.

Changes such as I have named were found in five post-mortem examinations of cases of diphtheritic paralysis by M. Déjerine as far back as 1878.* I have not been able to find any reference to this question of atrophy in diphtheritic paralysis as a characteristic feature of the disease in any of the authors whom I have consulted, and I am inclined to believe that it has not been much observed hitherto, because there is no reference to it in Dr. Neale's *Medical Digest* (last Edition). In the article "Diphtheria," in Quain's *Dictionary of Medicine*,† Sir J. Rose Cormack speaks of emaciation as a striking feature of the actual disease, and says that "the albuminous urine of diphtheria . . . is the expression of rapid waste of tissues;" but in his article, "Diphtheritic Paralysis,"‡ in the same work, this point is not referred to. I have already referred to the fact, with which every physician is familiar, that early and rapid atrophy is common in cases of spinal paralysis; but in the case of diphtheritic paralysis this feature is of special interest, both as throwing doubt on the theory that it is generally a paralysis of peripheral origin, and also as a clinical means of determining the *locus in quo* of the lesion. The most frequent spot of election for the development of morbid changes, judging from published cases, would appear to be in the medulla oblongata. The symptoms, as was long ago pointed out by Duchenne,§ will vary

* *British Medical Journal*, 1878, Vol. II., p. 477.

† Vol. I., 378. ‡ *Loc. Cit.*, Vol. II., 1098.

§ *Selections from the Works of Duchenne*, Poore's translation, p. 356.

according to the part of the medulla attacked. Thus in some, in fact in most cases, the paralysis is limited to the soft palate, and to this fact is probably due the theory, endorsed by Sir J. Rose Cormack, that diphtherial paralysis is peripheral in origin. On this point Duchenne observes: "The latter (palatal paralysis) might be looked upon as peripheral, and due to the inflammation of the region; but with it, or soon after, appear, but too often, other formidable symptoms which have not as yet received sufficient attention, and which, when carefully analysed, teach us, as I believe I have shown, that they are probably signs of the disease of the medulla oblongata." He proposes to call the affection *bulbar diphtheritic palsy*, and remarks of it:

"1. That it is symptomatic of disease of the medulla oblongata, principally the bulb.

"2. That the symptoms vary according as the nuclei are attacked successively, or together, or partially.

"3. That they are fugitive, but that many of the cases prove fatal."

All the cases I have quoted showed more or less involvement of the medulla, and the last probably proved fatal chiefly from implication of the spinal accessory nucleus.

Probably in the case of A. T. the very marked fall in the pulse rate which was observed was due even at that early stage to the action of the diphtheritic poison on the inhibitory fibres of the vagus. An interesting proof of this poisonous influence of diphtheria on the pneumo-gastrics is furnished by a case observed by Sir William Jenner, and quoted by the late Sir Thomas Watson,* in which the pulse fell to 32 and then to 24

* *Lectures on Principles and Practice of Physic*, 5th Edition, Vol. I., p. 895.

beats in the minute, the boy ultimately dying apparently of simple arrest of the heart's action.

Probably, then, in most cases of fatal diphtherial paralysis distinct organic changes will be found in the medulla, and sometimes also in the spinal cord. It is doubtless true that we get similar paralyses as sequelæ of typhoid and other acute infectious disorders, and also as a result of the introduction of certain poisons into the system. But it is, I think, no less true that we cannot any longer accept the opinion of Sir Thomas Watson that "we cannot suppose that the nervous symptoms—transient and wandering as they often are—can have their cause in any grave or material damage in the nervous centres."* The cases of Déjerine which I have quoted contradict this view, and doubtless as time goes on more will be added to our knowledge on this point.

*Postscript.*—Since this paper was written I have come upon a passage in the last edition of Dr. Hammond's work on *Diseases of the Nervous System* which bears upon the subject of the trophic centres, and which it may not be out of place to quote: "In admitting the existence of trophic cells in the anterior tract of grey matter of the spinal cord, I have been influenced by what I consider to be the weight of evidence. The fact is one which, in the present state of our knowledge, is not capable of absolute demonstration; but the subject is of such a character as scarcely to require proof of that nature. The inference for their existence is as strong as that which we draw relative to the presence in the spinal cord of groups of cells specially in relation with the functions of motion and sensation. As we have seen, there are affections of

* *Loc. Cit.*, p. 895. Trousseau expresses the same opinion in his *Clinical Lectures* (New Sydenham Society's translation), Vol. II., p. 565.

the cord in which there are both paralysis and atrophy. In such cases we have good reasons for concluding that the cells which are in nervous connection with the paralyzed and atrophied muscles have both motor and nutrient properties."*

With regard to the question of the peripheral or central origin of the paretic disorders, possibly the view of Dr. Senator might seem to be most in accord with the results of clinical observation. He remarks: "The few severe and fatal instances—those especially in which, by degrees, almost the whole of the muscular system becomes involved—impress one more with the idea of a central than of a peripheral paralysis. . . . On the other hand, it is difficult to say what may be the precise cause of those really peripheral paralyses which form the great majority."† I am inclined to think, however, that the distinction is somewhat arbitrary and artificial. In the case of E.W. the paretic condition of the lower limbs was so slight as almost to escape observation; but the knee-jerk was almost totally absent, showing some affection—transient, no doubt—of the lumbar portion of the cord; and I think that impairment or absence of the patellar reflex will be found in many cases in which, at the first glance, one would otherwise think the nervous disorder was limited to the medulla.

* *Diseases of the Nervous System*, 7th Edition, 1881, p. 533.

† *German Clinical Lectures*, 2nd series, p. 425. (New Sydenham Society.)

